# Nanoscale Drug Delivery Systems: From Medicine to Agriculture

**DOI:** 10.3389/fbioe.2020.00079

**Published:** 2020-02-18

**Authors:** Pablo Vega-Vásquez, Nathan S. Mosier, Joseph Irudayaraj

**Affiliations:** ^1^Laboratory of Renewable Resources Engineering, Department of Agricultural and Biological Engineering, Purdue University, West Lafayette, IN, United States; ^2^Department of Agricultural and Biological Engineering, Purdue University, West Lafayette, IL, United States; ^3^Department of Bioengineering, University of Illinois at Urbana-Champaign, Champaign, IL, United States

**Keywords:** drug delivery systems, nanotechnology, agriculture, encapsulation, phytonanotechnology

## Abstract

The main challenges in drug delivery systems are to protect, transport and release biologically active compounds at the right time in a safe and reproducible manner, usually at a specific target site. In the past, drug nano-carriers have contributed to the development of precision medicine and to a lesser extent have focused on its inroads in agriculture. The concept of engineered nano-carriers may be a promising route to address confounding challenges in agriculture that could perhaps lead to an increase in crop production while reducing the environmental impact associated with crop protection and food production. The main objective of this review is to contrast the advantages and disadvantages of different types of nanoparticles and nano-carriers currently used in the biomedical field along with their fabrication methods to discuss the potential use of these technologies at a larger scale in agriculture. Here we explain what is the problem that nano-delivery systems intent to solve as a technological platform and describe the benefits this technology has brought to medicine. Also here we highlight the potential drawbacks that this technology may face during its translation to agricultural applications, based on the lessons learned so far from its use for biomedical purposes. We discuss not only the characteristics of an ideal nano-delivery system, but also the potential constraints regarding the fabrication including technical, environmental, and legal aspects. A key motivation is to evaluate the potential use of these systems in agriculture, especially in the area of plant breeding, growth promotion, disease control, and post-harvest quality control. Further, we highlight the importance of a rational design of nano-carriers and identify current research gaps to enable scale-up relevant to applications in the treatment of plant diseases, controlled release of fertilizers, and plant breeding.

## Introduction

The potency and efficacy of an exogenously administrated bioactive molecule heavily depend on the extent of its prolonged availability in the intended final site of action. In turn, its availability depends on the intrinsic factors related to the nature of the molecule itself, such as its solubility (Savjani et al., 2012), pKa (Manallack, 2007), affinity for the receptor (Rang, 2006), molecular weight (Lajiness et al., 2004), among others. These characteristics largely influence the membrane permeability of the molecules and therefore, its capability to ingress to the target cell and produce its biological activity in it. On the other hand, some extrinsic factors such as the physiological stage of the receptor organism, enzymatic machinery, and external pH in the surrounding environment, make the drug prone to inactivation or degradation. Moreover, some other substances encountered throughout the organisms during the distribution process may interact with the drug in different ways resulting in either inactivation by the formation of molecular complexes, or either synergistic or antagonistic interactions (Foucquier and Guedj, 2015) which may modulate the potency of the drug or generate unexpected responses (FDA, 2012). After its administration, the processes of absorption, distribution throughout the circulatory system and subsequent metabolism may lead to physicochemical modifications due to the dynamic interaction with its new surrounding environment.

In order to successfully execute its therapeutic effect, a bioactive molecule must overcome every unfavorable physiological condition to reach its target in such a way that, a proper amount of active compound (i.e., adjusted within its therapeutic window) enters the target cell at a proper time. The challenge of drug delivery is to accomplish the release of the drug agents at the right time in a safe and reproducible manner, usually to a specific target site.

Drug delivery systems are engineered devices used to transport a pharmaceutical compound throughout the body in order to release its therapeutic cargo in a controlled manner (NIH, 2016). By encapsulating the molecules within a protective shell-like structure, potential physical-chemical or enzymatic disruptions of the active compound are diminished. In turn, not only the bioavailability of the active compound is increased but also undesirable side effects resulting from unspecific systemic distribution are reduced (Felice et al., 2014). Nano-encapsulation of bioactive compounds helps to reduce the frequency of dosing needed during treatment and also may confer physical protection to the drug during storage prior to its use for controlled release of cargo (Choudhury et al., 2017).

One of the most notable advantages offered by nano-delivery systems for drug therapy is the controlled drug release not only at a specific location level but in a time-dependent manner via passive or active targeting. Passive targeting drug nano-carrier is designed based on pathophysiological features from the targeted tissue that allow the accumulation of the nano-sized delivery system on it. On the other hand, active targeting refers to the coupling or assembly of surface-active ligands onto the surface of the drug delivery systems, which are able to recognize and interact with a receptor in the target cell. As a result of the interaction between ligands and receptors, the drug delivery specificity and nanoparticle up-take is enhanced (Felice et al., 2014). Different types of ligands have been successfully tested *in vitro* such as engineered antibodies, growth factors (Lee et al., 2010), vitamins (Chen et al., 2010), and aptamers (Colombo et al., 2015). Describing the complete pathway which had to take the controlled drug delivery systems from their very origins to their current state is not within the scope of this review. However, a highly detailed review describing the evolution of controlled drug delivery systems from their non-biodegradable macro-scaled state, up to the more updated biocompatible nano-carriers used in therapeutics is available (Hoffman, 2008).

The challenge of drug delivery is to accomplish the release of the drug agents at the right time in a safe and reproducible manner, usually to a specific target site. In this sense, medicine and agriculture share similar challenges and final goals. Similarly, nano delivery systems that have contributed to the development of precision medicine by delivering therapeutic molecules in a controlled manner have potential applications in agriculture. For instance, the use of encapsulated agrochemicals into nano-carriers to deliver pesticides to the desired crop to provide a focused delivery of the required dose (i.e., diminished application dosages), time-controlled release, and less eco-toxicity is not only an expanding area of research but a potential growth market (Slattery et al., 2019). Other areas within agriculture that could benefit from nano-encapsulation approaches include plant breeding (Kim et al., 2015), plant nutrition (Rai et al., 2015), growth promotion (Siddiqui and Al-Whaibi, 2015), disease control (Nuruzzaman et al., 2016), and post-harvest quality control (Yadollahi et al., 2010) to name a few. Conversely, agricultural materials such as cellulose (Bhandari et al., 2017, 2018) and chitosan (Cai and Lapitsky, 2019) have been used as base materials to develop drug delivery systems.

Nano-carriers intended for drug delivery can be prepared from a variety of materials such as proteins, polysaccharides, synthetic polymers and inorganic metallic salts (Panchapakesan et al., 2011; Wang et al., 2012). The selection of matrix materials depends on many factors such as the size of nanoparticles required; the physical properties of the drug (e.g., aqueous solubility and stability); the surface characteristics such as charge and permeability; the degree of biodegradability, biocompatibility and toxicity; drug release characteristics of the final product; and challenges involved in regulatory approvals. Scalability and approval from regulatory governmental entities are two other major concerns when the intention is to release a product to the market, which are closely related to the formulation and fabrication. The main objective of this review is to contrast the advantages and disadvantages of different types of nanoparticles and nano-carriers currently used in the biomedical field along with their fabrication methods to discuss the potential use of these technologies at a larger scale in agriculture. We also aim to highlight and discuss the applications of nano-encapsulation technology in agriculture and its potential drawbacks. Specifically, we address the use of nano-delivery systems as a non-viral vector for gene delivery in plant cells, and for the delivery of nutrients during plant growth promotion and crop protection.

## Nano-Drug Delivery Systems From the Engineering Perspective

Ideally, nano delivery systems should fulfill certain technical and economical requirements. Table 1 presents a summary of the characteristics of an ideal nano-carrier for biomedical and agricultural purposes. First, the materials used as carriers should not trigger any adverse response in the recipient organism. Also, not only the matrix material should be biocompatible, but its degradation products. Second, the mechanical properties of the polymer must provide prolonged protection to its cargo allowing chemical stability over time. Third, the scalability of the fabrication process should be technologically feasible and economically viable. Accordingly, the processes employed for the elaboration of nano-carriers should yield consistent results in a batch to batch basis, in terms of size, polydispersity, encapsulation efficiency, and stability. Finally, the materials to act as nano-carriers should be carefully selected since they not only must meet the technical criteria to address mandatory regulations prior to being commercialized (Tinkle et al., 2014), but they also must display good performance in terms of cost/benefit and eco-compatibility.

**Table 1:** Characteristics of an ideal nano-carrier for agricultural purposes.

**Fabrication conditions**	**Encapsulation properties**	**Release profile**
✓ Mild conditions ✓ Scalable ✓ Low-cost ✓ Reproducible ✓ Low batch-to-batch variability	✓Stable ✓ No early cargo release/leakage ✓ Non-toxic ✓ Biodegradable ✓ Eco-compatible Water soluble	✓Controlled ✓ Targeted ✓ Stimuli sensitive (pH, light, temperature)

Table 2 presents a summary of the advantages and disadvantages of drug delivery nano-carriers with potential use in agriculture. In general, drug nano-encapsulation depends on the physicochemical nature of the encapsulation matrix, the cargo, and the method to carry out the process. However, regardless of the encapsulation matrix and cargo nature; or the method used to fabricate the drug-loaded nano-carriers, a plethora of reports confirm that some processes to elaborate them have the potential to be standardized since their reproducibility is fairly consistent.

**Table 2:** Summary of advantages and disadvantages of drug delivery nano-carriers with potential use in agriculture.

**Type of nano-carrier**	**Advantages**	**Disadvantages**
Mesoporous silicon-based materials	Stable structure	Inorganic
	Tunable and uniform pore size	Non-biodegradable
	Controlled release of cargo	Potential cell lysis caused by silanol groups interacting with membrane lipids
Solid lipidic nanoparticles (SLNs)	Improves solubility in water of hydrophobic cargo	Low load capacity
	Hydrophilic cargo possible	Low Encapsulation efficiency
	Relatively inexpensive production	High water content in dispersions (70–99.9%)
	Biocompatible/biodegradable	Premature cargo release during storage
	Feasible production scaling-up	
Nano-emulsions	-Highly stable to gravitational separation and aggregation - Improves solubility in water of hydrophobic cargo - Biocompatible/biodegradable - Relatively inexpensive production - Suitable for incorporating lipophilic cargo - Increase efficacy of antimicrobial agents	- High amounts of surfactant needed to achieve oil droplets of nanometric sizes
Dendrimers	Functionalization of peripheral groups determines solubilization and enables targeted delivery of cargo	Cytotoxicity reported on cationic dendrimers
	Suitable for incorporating lipophilic or lipophobic cargo	Toxicity correlated with the number of surface amine groups
	PAMAM dendrimers are reported to be relatively resistant to hydrolysis	Pharmacokinetics, biodistribution, biodegradation, and chronic toxicity of PAMAM dendrimers are not yet clearly understood
Nanocrystals	Carrier-free (i.e., they are almost 100% drug)	Difficult to control morphology and crystallinity of final product
	Improves bioavailability of water-insoluble compounds Improves drug adhesiveness to surface cell membranes	Highly time/money/energy demanding. Need for large amounts of organic solvents (Bottom-up approach)
	Enhances particle stability in suspension	Residual presence of surfactants, solvents or stabilizers (top-down approach)
	Increase drug dissolution velocity	Specialized equipment is needed
Hydrogels	Complete bio and eco-compatibility Relatively inexpensive production Easy to fabricate	Batch to batch variation due to the heterogeneity of the polymer Fine tuning formulation required to achieve stable particles

## Nanoparticles and Nano-Carriers for Agriculture: Advantages and Disadvantages

### Metallic Nanoparticles

Due to their chemical nature, metallic nanoparticles such as gold and silver display enhanced physicochemical properties when presented as nanometric particles. Taking advantage of these properties, major efforts on research has focused on the development of devices, predominantly in the biomedical field, for detection and treatment. Chemical sensors are one of the most prominent biomedical applications of metallic nanoparticles (Guo and Irudayaraj, 2011). For instance, gold nanoparticles conjugated with specific oligonucleotides can sense complementary deoxyribonucleic acid (DNA) strands, detectable by color changes (Kouassi and Irudayaraj, 2006). Furthermore, gold nanoparticles can be readily functionalized with antibodies and oligonucleotides (Orendorff et al., 2006; Yu and Irudayaraj, 2007; Sun and Irudayaraj, 2009a,b; Wang et al., 2010; Wang and Irudayaraj, 2013;), enzymes (Majouga et al., 2015). These hybrid nanostructures are also active elements of a number of biosensor assays to detect gene products in plants (Kadam et al., 2014, 2017), drug and gene delivery systems (Ding et al., 2014). Although metallic nanoparticles are widely used in detection, these have limited applications as delivery systems.

### Mesoporous Silicon-Based Nano-Carriers (MPSNPs)

**Figure 1:**
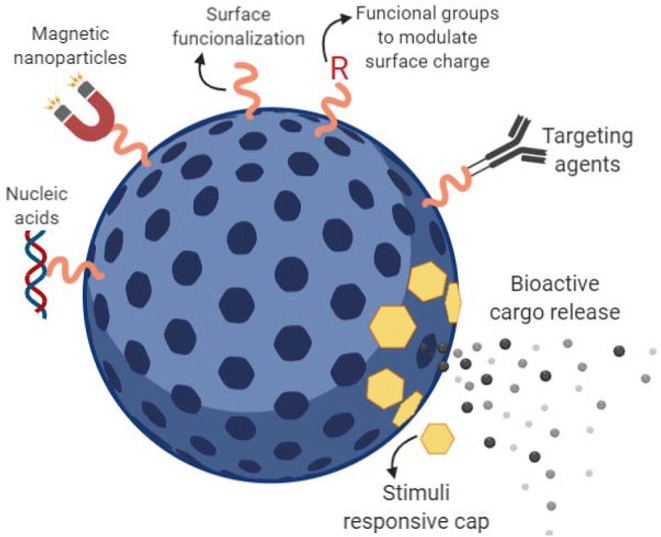
Mesoporous silicon-based nano-carriers (MPSNPs). Schematic representation of a mesoporous silicon-based nanocarrier. The bioactive cargo can be loaded into the porous spaces via passive adsorption or active anchoring. Stimuli responsive caps can be design to prevent early cargo release and detach from its pore allowing controlled release. Targeted cargo delivery can be performed by attachment of targeting agents onto previously functionalized particle surface.

Silicon-based mesoporous materials belong to the group of inorganic nano-carriers widely used as drug delivery systems. This approach takes advantage of the highly stable porous surface of silicon mesoporous materials to fill with bioactive cargo. Ideally, loaded pores are capped and the cargo is released intracellularly (Xu et al., 2019). One of the main advantages of MPSNPs is their stability, which confers the ability to cope with physical stress such as temperature and pH variations in their surrounding environment. Moreover, their tunable and uniform pore size (3–50 nm) allows them not only to load relatively high amounts of drug cargo due to their high surface area and large pore volume but to selectively functionalize candidate molecules onto its surface (Perez et al., 2017; See Figure 1). Different synthesis protocols to obtain fine-tuned large-pore mesoporous nano-carriers and their suitability in the delivery of proteins, enzymes, antibodies, and nucleic acids were explored (Knezevic, 2015).

The extended use of silicon-based mesoporous nano-carriers in clinical applications has been delayed due to the lack of pharmacokinetic-pharmacodynamic studies concerning biodistribution, clearance, therapeutic efficacy, and safety are important parameters that need further attention in the quest of providing competent porous nanoparticles (Shahbazi et al., 2012) For instance, it has been demonstrated that mesoporous silica nanoparticles is not completely hemo-compatible; such phenomena have been attributed to the surface density of silanol groups interacting with the surface of phospholipids or the red blood cell membranes resulting in hemolysis (Zhao et al., 2011). One of the potential drawbacks of the use of MPSNPs in agriculture is its non-biodegradability and lack of data on bioaccumulation to meet regulatory standards.

However, due to their intrinsic physico-chemical properties, the scope of use of MPSNPs include a wide range of applications such as: (i) water decontamination through adsorption of radioactive pollutants (Iqbal and Yun, 2018), separation of dyes (Shinde et al., 2017); (ii) catalysis (Verho et al., 2014; Munz et al., 2016); (iii) delivery of agrochemicals (Yi et al., 2015); (iv) chromatography (Ahmed et al., 2014) to mention a few.

### Solid Lipid Nanoparticles (SLN)

**Figure 2:**
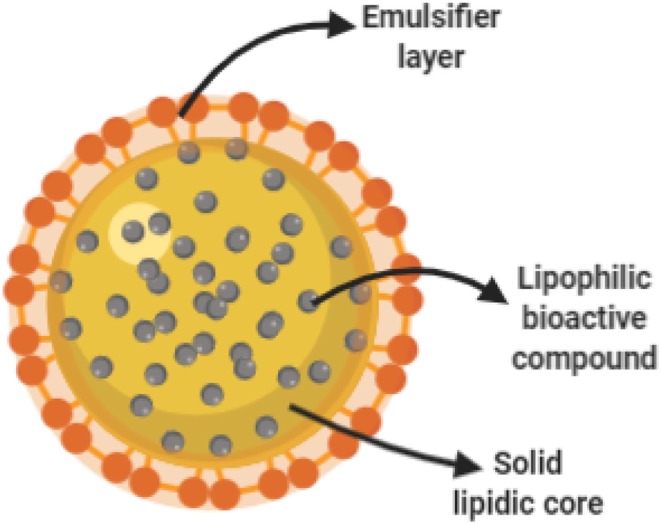
Schematic representation of a solid lipidic nanoparticle. During SLN fabrication, a lipophilic bioactive cargo is dissolved in a liquid hot lipid matrix. Under proper formulation and operational conditions, nanoparticles are formed assisted by an emulsifier as the lipidic core solidifies at room temperature.

Solid lipid nanoparticles (SLN) (Figure 2) are spherical nanoparticles, which makes these ideal candidates for the encapsulation of lipophilic bioactive compounds. The main advantage of SLN relies on their relatively low fabrication cost with the potential for scaling-up of production (Pallerla and Prabhakar, 2013). However, potential disadvantages for its use in agriculture include poor cargo loading capacity and early cargo expulsion after polymorphic transition during storage (Singhal et al., 2011; Pardeshi et al., 2012).

SLN have been successfully implemented in a wide range of applications. In the biomedical field, for instance, it has been used to increase both the solubility of several poorly soluble drugs, (Patel et al., 2012; Padhye and Nagarsenker, 2013) just to mention some. In the cosmetic industry, they have been used to encapsulate UV blockers such as 3,4,5-trimethoxybenzoylchitin (TMBC), 2-hydroxy-4-methoxybenzophenone and vitamin E for use as sunscreen (Wissing and Müller, 2001; Song and Liu, 2005). In the food industry, SLNs have been used to encapsulate antioxidant molecules such as ferulic acid and tocopherol (Oehlke et al., 2017), natural antimicrobial compounds (Piran et al., 2017), and hydrophobic flavoring agents (Eltayeb et al., 2013).

### Nano-Capsules

**Figure 3:**
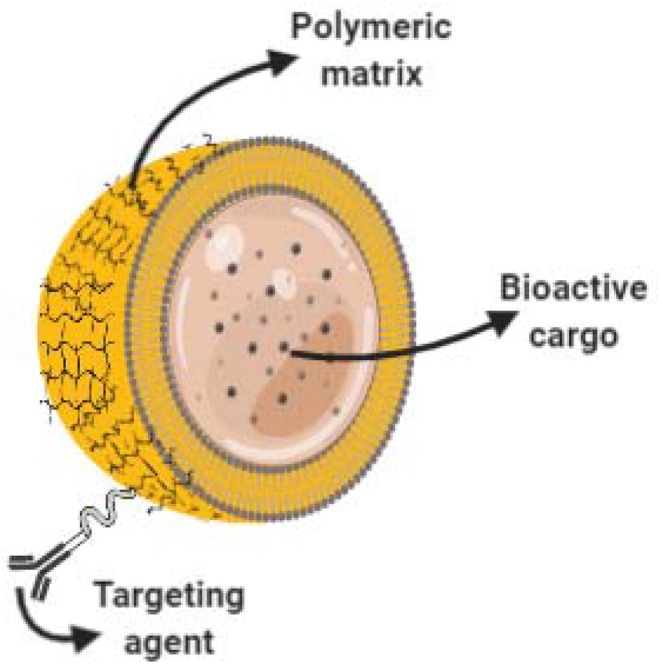
Core shell nano-capsules for drug delivery. Schematic representation of a nanocapsule. Bioactive cargo is encapsulated into a core-shell polymeric matrix. Polymer surface can be functionalized and decorated with targeting agents enabling targeted delivery.

Nano-capsules are nano-vesicular systems in which drugs are enclosed in an inner cavity created by a unique polymeric membrane (see Figure 3). Nano-encapsulation enhances drug delivery and efficacy, but the different methods used for the preparation of nano-capsules frequently produce dispersions with low drug loading. This is a serious disadvantage when the aim is to obtain therapeutic concentrations (Mora-Huertas et al., 2010). Similar to NLPs, the application of nano-capsules also extends from the pharmaceutical sector for the encapsulation and delivery of drugs, to the food industry and agriculture, as well as application in cosmetics and personal care in the form of cosmeceuticals.

Drug loaded nano-capsules are especially useful for skincare and dermatological treatments because of their enhanced bioavailability in dermal cells. Ebselen (Eb) is an example of a repurposed drug with poor aqueous solubility which requires a sophisticated delivery system such as nano-encapsulation for topical application as a promising, safe and complementary alternative to the treatment of cutaneous candidiasis (Jaromin et al., 2018). Examples of commercially available cosmeceutical products are “Hydra flash bronzer” a facial skin moisturizer, “Soleil soft-touch anti-wrinkle sunscreen,” “Soleil instant cooling sun” and “primordiale optimum lip” produced by Lancôme®. These products claim to contain nano-capsules of vitamin E and antioxidant agents as active ingredient. A more comprehensive list of readily available cosmeceuticals products containing nano-capsules and SLN is available in the literature (Lohani et al., 2014).

The food industry is taking advantage of the benefits of nano-encapsulating essential oils to enhance their antimicrobial activity against food borne-pathogens to increase their solubility when loaded into polymeric nano-capsules (Granata et al., 2018).

In agriculture, nano-encapsulation technology has been used for the delivery of currently available pesticide molecules (Yin et al., 2012). However, the increased water solubility, which is desirable for pesticide efficiency, brings environmental and in turn, regulatory concerns. By studying a commercially available insecticide with an encapsulated active ingredient, Slattery et al. demonstrated that by encapsulating the in nano-sized carriers, the active ingredient's water solubility increases. Enhanced water solubility disrupts foundational assumptions on its chemical behavior of the pesticide, such as its hydrophobicity (KOW) and soil sorption (Kd). The hydrophobicity (KOW) and soil sorption (Kd) values are numerical descriptors used to predict the environmental fate of a molecule (pesticide) and its toxicity. By encapsulating the pesticide molecules into nano-sized carriers, these indexes may not adjust to the prediction models once built based on their free un-encapsulated forms. Thus, complicating the use of hydrophobicity metrics to predict their fate and toxicity. Determining how carrier size influences the hydrophobicity (KOW) and soil sorption (Kd) of a given pesticide, and thus its mobility through soil and water, is important to our understanding of whether the current pesticide's toxicity risk assessments are sufficient to protect against products that incorporate nano-encapsulation technology (Meredith et al., 2016; Slattery et al., 2019).

### Micelles, Liposomes, and Nano-Emulsions

Micelles are spontaneously self-arranged spherical aggregates made of surfactant molecules. Liposomes are spherical vesicles with at least one lipid bilayer, and nano-emulsions are surfactant-assisted homogeneous suspensions of nano-sized droplets of a dispersed phase in a continuous phase. They all display spherical shape (Pavlic et al., 2009) and facilitated a controlled release of cargo (Godfroy, 2009; Joo et al., 2013). Besides their inherent biocompatibility, their surface can be modified and functionalized for conjugation with targeting moieties which enable targeting to specific sites, improving efficacy and potency (Vabbilisetty and Sun, 2014).

**Figure 4:**
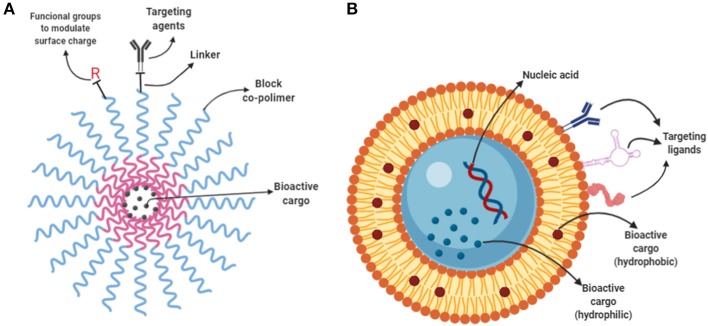
Polymeric micelles and liposomes. **(A)** Schematic representation of a polymeric micelles composed by a co-block polymer (red and blue wavy lines). The core shell is formed encapsulating the bioactive cargo inside. The surface can be functionalized with linker molecules and further decorated with targeting ligands to enable targeted delivery. **(B)** Depiction of a liposome containing hydrophilic cargo in its core a hydrophobic cargo allocated in the bilayer. Surface functionalization can be achieved by anchoring of targeting ligands such as antibodies, proteins and aptamers.

In general, liposomes (Figure 4B) are used to encapsulate water-soluble compounds because they are comprised of a lipid bilayer separating an aqueous internal compartment from the bulk aqueous phase. Whereas, oil in water (O/W) nano-emulsions are used to encapsulate hydrophobic compounds. In contrast, polymeric micelles (Figure 4A) are used to encapsulate both hydrophobic and hydrophilic compounds depending on the design. Block copolymers have a hydrophilic and a lipophilic block. Block-copolymers can easily reach NP size higher than 20 nm and close the liposomes.

**Figure 5:**
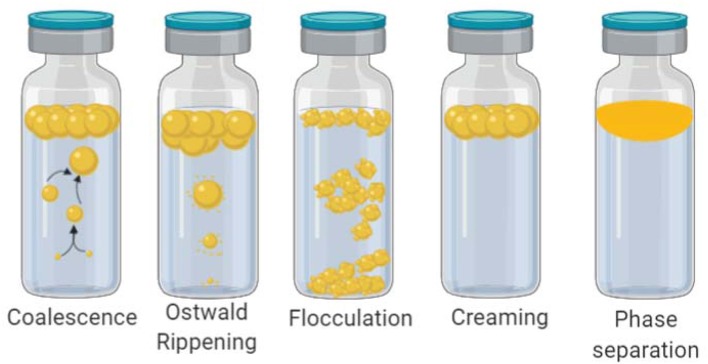
Types of emulsion destabilization. Schematic depiction of how emulsions naturally tend to separate its phases. (i) Coalescence occur when two separate oil droplets merge into a single larger oil droplet because surfactant monolayers fuse together. (ii) Ostwald ripening is the most common way of nano-emulsion failure. Larger oil droplets become larger at expense of smaller oil droplets driven by the pressure difference between to oil droplets of different diameters. The process accelerates as the diameter difference increases. (iii) Flocculation occurs when oil droplets collide, but instead of coalescence, they remain as independent droplets. Co-joined droplets form clusters that precipitate with enough time, the before mentioned processes produce (iv) creaming and later on they lead to complete (v) phase separation.

The applications of micelles, liposomes, and nano-emulsions include the encapsulation of poorly water-soluble bioactive molecules to be further incorporated into aqueous products. For instance, for biomedical purposes, a plethora of different types of drug-loaded nano-emulsions is available including oral, topical, intranasal and ocular administration (Yukuyama et al., 2017). In the food industry, several types of different nano-emulsions have also been used as carriers of natural occurring, but poorly soluble flavors, colors, preservatives and antioxidant agents (Donsì, 2018). Increased attention has been focused on the nano-emulsification of essential oils because it has been proven that when presented on a nanometric scale, their antimicrobial activity is enhanced. Moreover, its long-term stability is also enhanced (Figure 5). In a recent work D-limonene was used to prevent the formation of biofilms on *E.coli* O157:H7 at sub-lethal doses, by blocking the quorum sensing mediated autoinducer-2 (AI-2) communication and curli-related gene expression (Wang et al., 2018).

### Dendrimers

**Figure 6:**
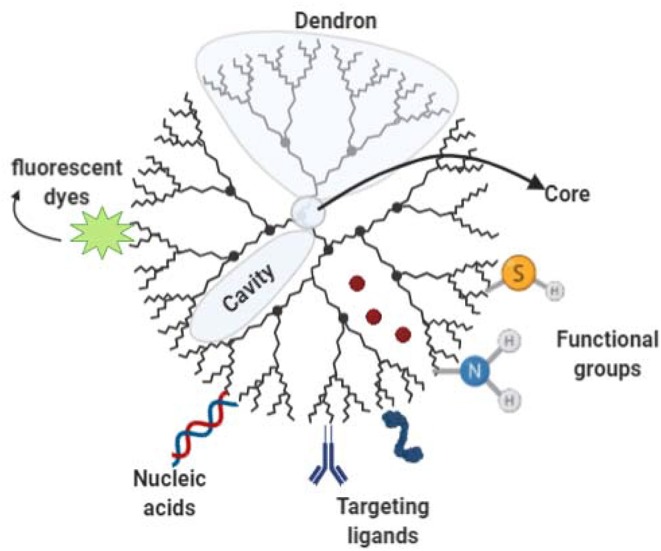
Dendrimer structure and functionalization Schematic representation of a Dendron comprising Dendron units branching out of a focal core interspaced by cavities. Bioactive cargo can be encapsulated into cavities. Dendron ends can be functionalized allowing targeting ligand attachment, fluorophore molecules, nucleic acids among other molecules of interest.

Dendrimer structures are comprised of three components (Figure 6): a focal core, dendrons, and cavities formed between dendrons (Safari and Zarnegar, 2014). Some of the desirable characteristics of dendrimers are their uniform molecular weight and their three-dimensional structure with peripheral groups that determine solubility, making them relatively easy to design upon specific demands. Further, their smaller hydrodynamic volume and lower molecular volume compared with linear polymers of similar molecular weight (Markowicz-Piasecka and Mikiciuk-Olasik, 2016). Exposed terminal groups in dendrimeric particles mostly control their chemical interactions with the molecular environment. Their properties such as nanometer size range, ease of preparation and functionalization, also their multiple copies of surface groups displaying stability, make them an attractive system for drug delivery. However, despite their initial popularity in drug delivery, at present, serious concerns exist on the cytotoxicity of cationic dendrimers, which has led to further investigation of alternatives to overcome this issue. The toxicity of dendrimers mainly comes from the high cationic charge density in the periphery, where charges interact with the biological cell membrane and then result in membrane disruption (Tsai and Imae, 2011).

Dendrimer-based non-viral vectors for gene delivery have gained traction over the past two decades, especially in the field of biomedicine for cancer treatment. In plants, the use of cationic polyamidoamine (PAMAM) vector assisted by ultrasound has been used for DNA delivery. Amani et al. (2018) demonstrated in alfalfa cells, that single and double-stranded DNA transfection efficiency can be significantly improved when PAMAM dendrimers are used assisted by sonication (Amani et al., 2018). Production of dendrimers can be approached in two different ways: convergent approach and divergent approach, each with its own limitations (Gupta and Nayak, 2015). Although the main applications of dendrimers are in drug/gene delivery for biomedical applications (Palmerston Mendes et al., 2017), several other applications exist (Abbasi et al., 2014).

Dendrimers are also useful for agricultural purposes. They may improve the delivery of agrochemicals intended to either promote growth or discourage diseases. For instance, in 2016, a crop protection company (Adama) licensed Starpharma's (ASX:SPL) Priostar dendrimer technology for the development of an enhanced 2,4-D herbicide for the US market. According to the manufacturer, some of the potential benefits from the use of dendrimer technology in crop protection include improved efficacy, more concentrated formulations to reduce transport costs, reduction in solvent requirements and increased adhesion. Stapharma's lysine dendrimer based Vivagel® managed to achieve clinical approval (Moura et al., 2019) which indicates the technological feasibility of mass-production. However, the technical details regarding up-scaled production are not publicly available.

The use of dendrimers for crop protection faces the challenge impose by mass-production. The main challenge relies on preserving their purity and monodispersity upon up-scaled manufacture. Technical details with respect to improved reaction conditions and purification of half- and full-generation PAMAM dendrimers to overcome the critical limitations for upscaling this class of polymers are available elsewhere (Ficker et al., 2017).

### Nanocrystals

Nanocrystals are another nanotechnological approach to deliver poorly soluble drugs. In contrast to the prior mentioned drug delivery system platforms, nanocrystals have several unique traits. Drug delivery nanocrystals are carrier-free colloidal delivery systems (i.e., they are almost 100% drug). Thus, drug nanocrystals possess the merits of improving the oral bioavailability of water-insoluble compounds, reducing administered dose, avoiding abnormal absorption thus minimizing utilization of large excipients, increasing dissolution velocity, increasing adhesiveness to surface cell membranes, and increasing particle stability (Wang et al., 2011). Conventionally, drug nanocrystals can be produced whether from a top-down or a bottom-up approach. The demand for energy, time and money is high for top-down approaches such as milling or high-pressure homogenization. For instance, high-pressure methods require specialized equipment able to deliver up to 1,700 bar for over 100 homogenization cycles, and the milling method requires hours if not days to achieve the desired particle size, depending on the drug properties (Lu et al., 2016). Moreover, the grinding process may contaminate or denature labile drugs which may lead to unexpected side effects on the recipient patient. Further concerns exist on the potential loss of bioactivity and molecular integrity due to severe thermogenesis derived from the milling process. Other disadvantages for the top-down methods are: The lack of complete control of the morphology and crystallinity of the final product; particle aggregation/agglomeration issues; losses of the product due to drug adherence to equipment surfaces and residual presence of surfactants, solvents or stabilizers (Padrela et al., 2018).

In comparison, bottom-up processes are achieved through nucleation and subsequent crystallization. One way to achieve nucleation is by mixing the drug with an antisolvent by simple stirring. Another way is to remove the solvent via spray and freeze-drying. Subsequent crystallization does require high energy methods such as sonication or intense micro-stirring (Lu et al., 2016). Another approach to producing drug nanocrystals is based on supercritical carbon dioxide (ssCO_2_). The details on the roles of ssCO_2_ as solvent, co-solvent and as an additive for the production of drug nanocrystals are comprehensively reviewed elsewhere (Padrela et al., 2018). Amongst the main disadvantages of the bottom-up methods to produce drug nano-crystals are: (i) the difficulty to control the particle size, nucleation, and growth of crystals that may lead to both, undesired morphologies or amorphous crystallinities and subsequent particle agglomeration; (ii) the need for large amounts of organic solvents; (iii) fine-tuning solvent/antisolvent formulation is time-consuming; (iv) need for solvent removal; (v) labile drugs may denature during heating solvent removal; (vi) need for specialized equipment for ssCO_2_-based nanocrystals (Padrela et al., 2018).

The use of nanocrystals in agriculture has enormous potential for sustained and efficient nutrient delivery into crops. For instance, nitrogen can be applied in the form of Urea-Hydroxyapatite nanohybrids. When tested in rice fields, urea-hydroxyapatite nanohybrids significantly enhanced nitrogen bioavailability, resulting in higher crop yields, while reducing the nitrogen input up to 50%, when compared to granular urea (Kottegoda et al., 2017). The efficacy of hydroxyapatite nanoparticles as Phosphorus fertilizer has also been studied in andisols and oxisols. Montalvo et al. showed that the effect of phosphorus in the form of hydroxyapatite nanoparticles, in the wheat dry matter production significantly depends on the type of soil these particles are applied on. Nano-Hydroxyapatite in strongly phosphorous sorbing soils had more effect on shoot dry matter production and plant phosphorous uptake than bulk-HAP but less than the water-soluble triple superphosphate. This is maybe due to the propensity of nano-hydroxyapatite to aggregate, thus reducing both the mobility and the dissolution rate of the particles (Montalvo et al., 2015). Since nano-nutrient/soil particle interaction is strongly affected by the intrinsic heterogeneity of the soil, it is reasonable to study alternative nano-nutrient up-take pathways in plants. In a recent study, Avellan et al. analyzed how nano-crystals move throughout the plant, from the leaves to the roots, using gold nanoparticles as a model in wheat. They found that “regardless of their coating and sizes, the majority of the transported AuNPs accumulated in younger shoots (10–30%) and in roots (10–25%), and 5–15% of the NPs <50 nm were exuded into the rhizosphere soil. A greater fraction of larger sizes AuNPs (presenting lower ζ potentials) was transported to the roots” (Avellan et al., 2019).

Accounting for these disadvantages, scaling up of its production has been a challenge. It is also worth noting that there is a lack of cytotoxicity studies, and the details of the intracellular fate of the nanocrystals are not well-understood (Junyaprasert and Morakul, 2015).

### Nanogels

Nanogels are hydrophilic cross-linked networks forming polymer chains that absorb substantial amounts of aqueous solutions. Due to their conformational tridimensional structure, hydrogels are capable of imbibing bioactive molecules solubilized in water or aqueous fluids. The presence of chemical crosslinks (tie-points or junctions) or physical crosslinks, such as entanglements or crystallites, are responsible for their characteristic conformational structure and size (Himi and Maurya, 2013), which can be fine-tuned via chemical control of the formulation and the process to obtain the hydrogel nanoparticles. The main advantages of hydrogels, when used as drug delivery systems, is their complete biocompatibility due to their high content of water (Caló and Khutoryanskiy, 2015). On the other hand, one of the major drawbacks of these types of particles is the batch-to-batch variation due to the heterogeneity of the polymer itself, such as the case for chitosan-based drug delivery systems.

**Figure 7:**
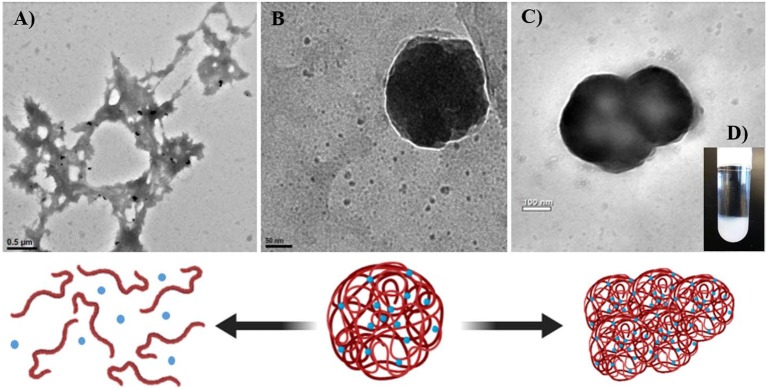
Factors influencing formation and stability of chitosan-based nanoparticles mediated by the cross-linker tripolyphosphate (TPP). Formation and stability of chitosan-based nanoparticles are sensitive to formulation and preparation conditions. **(A)** When the amount (per mole) of cross-linker (TPP) is insufficient relative to the amount (per mole of ${\text{NH}}_{3}^{+}$ from chitosan), chitosan particles **(B)** rapidly dissolve at pH levels below its pKa. When the pH of the solution is not acid enough, amino groups from chitosan deprotonate preventing chitosan to dissolve and then failing to form electrostatic interactions with the crosslinker, resulting in particle dissolution and ulterior precipitation. **(C)** Excess of crosslinker in the solution result in particle aggregation and **(D)** further precipitation.

Coacervation or ionic gelation method is one of the most common processes carried out to produce this type of nanoparticles because it is easy to implement and requires un-expensive materials. In general, the process involves the mixture of two aqueous phases, where one of which is the polymer and the other is the dissolved cross-linker. It is common to use an oil/water emulsion as one of the aqueous phases containing the bioactive hydrophobic molecule or drug of interest to be encapsulated within the forming capsule. The method is relatively easy to perform since it does not require sophisticated equipment, which is imperative for the scaling up. However, the final characteristics of the produced nanoparticles, such as size, polydispersity, and stability, are highly sensitive to changes in the fabrication conditions, such as pH, ionic strength, stirring speed, addition rate, and type and concentration of polymers and cross-linkers. Chitosan-based nano-carriers are of special interest in agriculture. However, the literature regarding both, nanoparticle formation via ionic gelation and cargo release profile, is overwhelmingly inconsistent (Huang et al., 2015; Cai and Lapitsky, 2019). An example is illustrated in Figure 7 showing factors that influence chitosan nanoparticles' formation and stability.

Typical applications of hydrogels revolve around the biomedical field, including drug encapsulation, transport and delivery; tissue engineering for wound-healing treatment; and 3-D cell culture. Nonetheless, hydrogels can also be used as antimicrobial agents. Chitosan, for instance, is a polymer commonly used to fabricate nano-carriers, naturally displays antimicrobial activity. Metal ions, such as Ti3^+^, Fe3^+^, Ag^+^, Cu2^+^, and Zn2 can also be incorporated into non-antimicrobial hydrogels in order to confer antimicrobial properties. Incorporation of some metallic ions can also confer catalytic, photo-responsive, photochemical, redox, and conductive properties to hydrogels (Wahid et al., 2017). In agriculture, the use of chitosan nanoparticles are of special interest due to its immune-modulatory activity elicited in plants. Chitin is a pathogen-associated molecular pattern (PAMP), detected by a transmembrane chitin receptor (LysM/CERK1) in plant cells. Sensing chitosan triggers an intracellular defense immune response (i.e., PTI—Pathogen triggered immunity) involving the activation of kinases and up-regulation of defense-related genes, such as plant defensin PDF1.2, resulting in jasmonic acid and ethylene accumulation associated with immunity to necrotrophs (Mengiste, 2012; Malerba and Cerana, 2016).

## Drug Delivery Systems in the Agriculture

**Figure 8:**
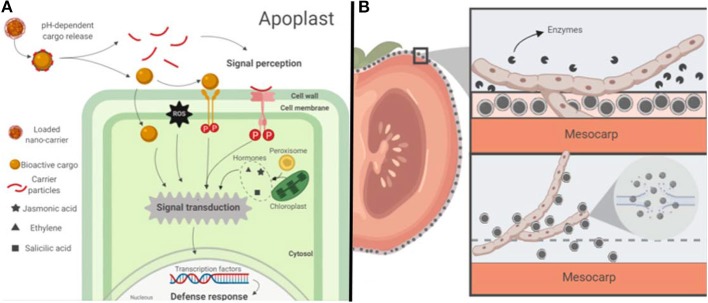
Schematic representation of the potential mode of action of drug-nanocarriers applied in the agriculture: **(A)** Example of a potential mode of action of drug nano-carriers for systemic protection of plants: A pH-sensitive polymeric loaded nano-carrier enters the plant apoplast and releases its cargo. The bioactive payload may enter to the plant cell cytosol or Activate a signaling cascade upon recognition by a transmembrane receptor triggering the plant defense immune response. Carrier molecules (e.g., chitosan) can also elicit an immune response in plants upon recognition by receptors. **(B)** Example of a potential mode of action of drug nano-carriers for post-harvest produce protection: (a) Fungal extracellular enzymes degrade an edible coating with embedded nano-carriers. (b) Drug nano-carriers loaded with antimicrobial compounds are released from the coating. Fungal membrane is disrupted by direct contact with antimicrobial compounds.

According to the United Nations, the estimated world population projected for 2050 will be 9.7 billion people. The increasing world population brings challenges that may imbalance the food production chain at various levels such as social, economic, technologic and environmental. Efforts to find new strategies that will allow improving the quantity and quality of food supply under a scheme of sustainability are imperative to meet the demands of the incoming population. The application of engineered nano-carrier devices, intended for the delivery of encapsulated molecules, could be a promising alternative to meet the future agriculture needs of increased productivity. Phytonanotechnology (i.e., the application of nanotechnology in plants) may improve the way we grow crops. Nano-delivery systems enable the controlled release of agrochemicals (e.g., fertilizers, pesticides, and herbicides) and target-specific delivery of biomolecules (e.g., nucleotides, proteins, and activators; Wang et al., 2016; see Figure 8, Table 3).

**Table 3:** Some commercial product of nanofertilizers.

**Commercial product**	**Content**	**Company**
Nano-Gro™	Plant growth regulator and immunity enhancer	Agro Nanotechnology Corp., FL, United States

Nano green	Extracts of corn, grain, soybeans, potatoes, coconut, and palm	Nano Green Sciences, Inc., India

Nano-Ag answer®	Microorganism, sea kelp, and mineral electrolyte	Urth Agriculture, CA, United States

Biozar nano-fertilizer	Combination of organic materials, micronutrients, and macromolecules	Fanavar Nano-Pazhoohesh Markazi Company, Iran

Nano max NPK fertilizer	Multiple organic acids chelated with major nutrients, amino acids, organic carbon, organic micro nutrients/trace elements, vitamins, and probiotic	JU Agri Sciences Pvt. Ltd, Janakpuri, New Delhi, India

Master nano chitosan organic fertilizer	Water soluble liquid chitosan, organic acid, and salicylic acids, phenolic compounds	Pannaraj Intertrade, Thailand

TAG NANO (NPK, PhoS, Zinc, Cal, etc.) fertilizers	Proteino-lacto-gluconate chelated with micronutrients, vitamins, probiotics, seaweed extracts, humic acid	Tropical Agrosystem India (P) Ltd, India

*Source: Ram Prasad, Atanu Bhattacharyya et al. Frontiers in Microbiology, 8, JUN, 6 2017 (creativecommons.org/licenses/by/4.0)*.

Nano-encapsulated pesticides offer enhanced controlled release of cargo and enhanced efficacy. Regarding the development of nano-pesticides is worth noting that a common practice in this industry is to focus on the modification of already registered existing molecules, rather than discovering new molecules. This is due to the costs associated with the development and further registration which is a process often measured in years. A commercially available capsule suspension insecticide (Environmental Protection Agency (EPA) Reg. No. 67760-104-53883) with 5.9% γ-cyhalothrin; and an EPA registered capsule suspension insecticide with 22.8% λ-cyhalothrin (EPA Reg. Number 100–1,295, Greensboro, NC, USA) are two examples of nano-pesticides currently available in the market under this reformulation scheme (Meredith et al., 2016; Slattery et al., 2019).

### Nano-Carriers as a Non-viral Vector for Gene Delivery in Plant Cells

In order to obtain higher crop production yields, it is necessary to develop new plant varieties by introducing traits that ideally enables them to better resist different environmental-derived abiotic stresses or pathogen-mediated diseases along with the generation of higher biomass under limited resources. The transfer of genes to the target plant cells is challenging due to the rigid plant cell wall which prevents the exogenous particle movement from the outside to the cytoplasm (Abd-Elsalam and Alghuthaymi, 2015). There is evidence that the plant's nano-particle uptake is strongly dependent on the cell wall pore diameter (i.e., exclusion size limit), which may vary amongst different tissues and organs. In general, the plant cell wall's exclusion size limit is up to 50 nm (Cunningham et al., 2018). Due to its small size, nanoparticle-enabled gene delivery into plant cells pose a promising option for genetic engineering for agriculture. The first reported example of this was done by Torney et al. who managed to develop a 3-nanometer pore mesoporous nanoparticle (MSN) able to transport DNA and chemicals into isolated plant cells that interact with leaves. MSN were designed in such way that gold nanoparticles capped the pores in order to avoid cargo leakage and release the content in the intended target to trigger gene expression under controlled-release conditions (Torney et al., 2007).

For plant genetic recombination purposes, exogenous gene delivery into plant cells is required. In animal models, nanoparticle penetration into cells is often reported to be improved when mediated with ultrasound. Ultrasound-assisted gene delivery is in use for plants because of its easier operation, lower cost and no plant specificity constraints among others (Liu et al., 2005). Nevertheless, the main disadvantage of ultrasound-mediated technique is that naked DNA is highly sensitive to external high energy sources and as a result, it may suffer damage, especially when increasing ultrasonic strength and time to achieve high transfection efficiency; so the ultrasound-mediated transgenic method has been largely restricted in practice (Yu-qin et al., 2012). Interestingly, DNA-nanoparticle complexes can protect DNA from ultrasound damage as well as from enzymatic degradation (Liu et al., 2005). DNA-nanoparticle complexes that have been studied before included Zinc and Calcium phosphate (Naqvi et al., 2012; Yu-qin et al., 2012).

Foreign particle uptake in plants can naturally occur either via endocytosis or by direct penetration. In plants, different engineered nanomaterials can be used for nanoparticle-mediated DNA transfer using gene-nanoparticle (NP) anchoring using zinc, calcium phosphate, silica, gold, magnetite, strontium phosphate, magnesium phosphate, and manganese phosphate (Sokolova and Epple, 2008; Mahendra et al., 2012) and carbon-based materials such as starch (Sun et al., 2009) fullerenes, single-walled carbon nanohorns (SWCNHs), single-walled carbon nanotubes (SWCNTs), multi-walled carbon nanotubes (MWCNTs) (Burlaka et al., 2015) and dendrimers. However, it has been reported that nanoparticle uptake by plant cells undergoes faster when positively charged nanoparticles are used rather than negatively charged nanoparticles, perhaps due to the preference of the negatively charged cell wall for cations (Cunningham et al., 2018).

Chitosan-based nano-carriers are a promising platform for cargo delivery into plant cells because it is positively charged, amongst other advantages it has. A recent study demonstrated organelle-targeted delivery and transient expression of genetic material via chitosan-complexed single-walled carbon nanotube carriers. Successful transformation of chloroplasts was achieved in mature *Eruca sativa, Nasturtium officinale, Nicotiana tabacum*, and *Spinacia oleracea* plants and in isolated *Arabidopsis thaliana* mesophyll protoplasts.

Since the plastid genome is maternally inherited in most plants, organelle-specific gene delivery is important because it can prevent the potential proliferation of genes to weedy relatives (Kwak et al., 2019). In this specific study, the authors showed that chitosan-complexed single-walled carbon nanotubes (SWNTs) uptake mechanism was described by the lipid exchange envelope penetration (LEEP) model, whereby the ability of nanoparticles to penetrate the cell membrane and the chloroplast envelope is governed primarily by the nanoparticle size and surface charge (Kwak et al., 2019)

In conclusion, nanoparticle assisted gene delivery systems initially developed for medical purposes has been shown to display the same delivery function in plant cells. It is worth noting that the individual performance of DNA delivery into plant cells must be evaluated on a case to case basis since the results presented in literature has several inconsistencies related to the transformation efficiencies achieved by different materials on different plant models. However, the evidence suggests that the concept of non-viral gene delivery into plant cells is promising. The specific design of nanoparticles should respond to the specific demands of the plant model/gene to be transferred, therefore no universal or generic delivery system for gene delivery into plants has been developed.

### Nano-Delivery Systems for Nutrition and Growth Promotion in Plants

Commercial fertilizers play a critical role in improving crop yields, however, inherent inefficiencies derived from the nature of the soil, plant health, environmental conditions, or the fertilization method among other factors, can lead to dire negative economic and environmental consequences that may endure in the long term. Not all the nutrient ions in fertilizer applied to a field soil are uptaken by the growing crop. At least three things can happen to the remaining residues from chemical fertilization: They may persist in the soil or, washed away by water leaching through the soil either downwards or throughout the surface or, lost to the atmosphere by volatilization.

In particular, higher than optimum nitrogen, phosphorus and potassium levels can lead to excessive plant and algal growth in waterways that can degrade potable water, fisheries, and recreational areas; leach nitrates into underground or sea waters and release nitrogen-oxides into the atmosphere. Phosphorous losses are also a major environmental concern derived from excessive fertilization in agriculture. It is estimated that the overall efficiency of applied phosphorus to the soil is <20% (Balemi and Negisho, 2012). Nutrient depletion leads to a variety of plant symptoms which affects the overall yield of a crop. Similarly, over-fertilization leads to an ecological imbalance which is hard to restore. Excessive soluble salts from fertilizers alter soil salinity, which in turn alters the soil pH; lower pH values diminish the availability of nutrients to plants by causing an imbalance in the soil native microbial ecology, responsible for nutrient solubilization.

Excessive fertilization is common due to soil nutrient heterogeneity. Overfertilization releases to the environment nutrients that cause, for instance, eutrophication of water bodies. Estimated losses of nitrogen, phosphorus and potassium are around 40–70%, 80–90%, and 50–90%, respectively. In a practical scenario, very less concentration (much below to minimum desired concentration) reaches the targeted site due to leaching of chemicals, drift, runoff, evaporation, hydrolysis by soil moisture, and photolytic and microbial degradation losses. Thus, nano-delivery systems for controlled release emerge as a highly valuable technology with the potential to strengthen the responsive capabilities of a sustainable food chain supply.

The application of nanotechnology for fertilizer delivery is encouraging. Patent applications related to nano-fertilizers are growing consistently according to the world intellectual property organization database. a 10% increment in patent filings related to nano-fertilizers from China, in a period of fewer than 3 years (01/2014–11/2016). This is consistent with data reported by Mastronardi et al. (2015) who noted a 10x (c.a) increase in patent results (Ref. SciFinder) over a 10 year period from 2002 to 2012 (Mastronardi et al., 2015). Current applications of nanotechnology in fertilization and plant protection can be divided into three different categories: (1) Nanoscale fertilizer inputs, which describes examples of nano-sized reformulation of fertilizer input in such a way that the size of the fertilizer or supplement is reduced down to nano-scale. (2) Nanoscale additives, which include the additives presented as nanoparticles and added to bulk materials, and (3) nanoscale coatings or host materials for fertilizers, which includes nano-thin films or nanoporous materials used to encapsulate fertilizers for the controlled release of nutrients in crops (Mastronardi et al., 2015).

Current applications of nanotechnology in fertilization and plant protection can be divided into three different categories (1) Nanoscale fertilizer inputs, which describe examples of nano-sized reformulation of fertilizer input in such a way that the size of the fertilizer or supplement is reduced down to the nano-scale. (2) Nanoscale additives, which include the additives presented as nanoparticles and added to bull materials. And 3, nanoscale coatings or host materials for fertilizers, which include nano-thin films or nanoporous materials used to encapsulate fertilizers for the controlled release of nutrients in crops (Mastronardi et al., 2015). Gao et al. working with spinach, have shown an enhancement of plant growth when titanium dioxide nanoparticles (TiO2-NPs) were administered to the seeds or when they were sprayed onto the leaves. TiO_2_-NPs were shown to increase the activity of several enzymes and promote the adsorption of nitrate, which accelerated the transformation of inorganic nitrogen into organic nitrogen (Gao et al., 2008). The current understanding of the mechanisms involved in nanoparticle uptake and translocation from leaves to roots were discussed earlier in this document (see section Nanocrystals).

### The Importance of Nano-Delivery Systems for Disease and Pests Control in Crops

In 1985, Pimentel and Levitan reported that approximately 500 million kilograms of pesticides were applied to plants in the United States (U.S) each year, but only 0.1% of this reach its desired target to effectively eliminate pests (Pimentel and Levitan, 1986). Over 25 years later, in 2011, Pimentel and Burgess reinforced this statement, stating that 545 million kilograms of pesticides were applied to crops in the United States each year, and several applications show that <0.1% of these pesticides reach their target (Pimentel and Burgess, 2012). The use of pesticides including herbicides, insecticides, and fungicides is consistently increasing worldwide, but nowadays, we do not know exactly by how much. According to the United States Department of Agriculture (USDA), the total pesticide expenditures in U.S. agriculture reached close to $12 billion in 2008, a 5-fold increase in real terms (adjusted for inflation) since 1960, but well-below the $15.4-billion peak reached in 1998 (Fernandez-Cornejo et al., 2014). The most recent report about pesticide usage dates to 2017 covering data from 2008 to 2012. According to the report, by 2012 over an estimated 380,000 tons were used in the US, from which 282,000 tons were herbicides, with a total expenditure over 9 billion $US (Atwood and Paisley-Jones, 2017). The lack of up-dated data reports in this regard makes it difficult to enable an informed pesticide policy debate, as well as sway science-based decisions in the right direction.

It is conceivable that improving the targeting and accuracy of pesticides could substantially reduce the amount of toxic chemicals that are applied to crops and improve the yield and safety of agriculture. Ideally, a pesticide should be able to remain active regardless of the environmental conditions in order to perform its intended biocide action. Correspondingly, it should also overcome the defense mechanisms from the pest it must target, it should also be harmless to the surrounding flora and fauna, and be engineered in such a way that it can be mass-produced at the lowest possible cost in order to guarantee economic returns to farmers. Current pesticides fail to completely fulfill these requirements, which results in more frequent and higher doses application schemes, and therefore, higher economic, and environmental costs. Nanomaterials used as a pesticide or as a carrier material have exhibited functional properties such as stiffness, permeability, crystallinity, thermal stability, and biodegradability over commonly used pesticides (Bordes et al., 2009).

Increasing wealth of knowledge in the literature regarding the development and use of pesticide-loaded nano-carriers intended for crop protection supports the importance of this technology toward sustainable agriculture by increasing the potency and bioavailability of pesticides, thus reducing the total amount of agrochemicals released in the environment. Pesticides such as β-cypermethrin (an insecticide; Wang et al., 2007), tebuconazole (a fungicide; Díaz-Blancas et al., 2016), and atrazine (a herbicide; Oliveira et al., 2015) presented as nano-encapsulated formulations are some examples of the potential use of nano-carriers to enhance the biological activity of active ingredients and also increase their stability over time. Zhao et al. (2017) demonstrated that it is feasible to develop a nano-emulsified pesticide displaying not only high stability over time (90 days) but also stronger absorption on negatively charged surfaces, which are desirable characteristics for spray-based foliar applications of pesticides in crops (Zhao et al., 2017).

## Prospects of Nano-Delivery System Technology in Agriculture

Based on the data collected from the literature, we expect at least two main positive impacts of the extended, prolonged and improved use of nano-delivery technology translocated into the food production chain. The first is related to the technical aspects of pesticide usage. Similar to the role they play in the medical field, nano-delivery systems can increase the controlled-release properties of the pesticide, increased solubility of active ingredients, protection against premature degradation and increase the stability of active ingredients. Another advantage is that non-target surrounding or distant flora and fauna will be less affected as a result of reducing exposure to toxic chemical compounds. In addition, the technical constraints concerning the massive production of nano-carriers for use in agriculture should be correlated with the economical boundaries which limit the production costs and configures the potential revenues for producers. Additional studies are required to assess, not only the fate of nano-encapsulation materials and payloads, and the resulting physical-chemical and biological performance, but the long-term environmental risks and economic viability.

## Conclusions

It is clear that there is an immense need to develop methods or technologies that allow us to cope with the contrasting challenges of the food supply chain. For instance, the toxicity threshold of materials used in the delivery system is species-dependent and responses to these are driven by a series of factors including not only the nanomaterial itself but the environmental and physiological conditions on which they are applied. Another noteworthy factor is the broader impact of the delivery system to the environment, while in the medical systems, it is localized to the individual receiving the treatment. Impacts on plant growth, and therefore on product yield and food quality, have been reported. However, several gaps exist in understanding the dynamics of interactions between plants and engineered nanomaterials (ENMs). Given the lack of experimental standardization and the divergent responses, even within similar plant species, it is challenging to foresee the challenges on the use of ENMs in plants (Zuverza-Mena et al., 2016). Finally, there is an imperative need to standardize and validate protocols to assess the positive and negative impact of nano-carriers in an experimental setting, and scale-up of testing can yet be another challenge. Most of the currently available information stems from experiments under controlled conditions, making it difficult to predict the real potential of functional prototypes. Research efforts could focus on controlled release, particle stability, and environmental fate and toxicity to make this a fully-embedded technology.

## Author Contributions

The initial framework and idea were conceptualized by PV-V and JI. All authors contributed toward the writing of this article.

### Conflict of Interest

The authors declare that the research was conducted in the absence of any commercial or financial relationships that could be construed as a potential conflict of interest.
